# Development of a video-assisted thoracoscopic lobectomy program in a single institution: results before and after completion of the learning curve

**DOI:** 10.1186/s13019-016-0526-8

**Published:** 2016-08-05

**Authors:** Alessandro Gonfiotti, Stefano Bongiolatti, Sara Borgianni, Roberto Borrelli, Massimo O. Jaus, Leonardo Politi, Giorgia Tancredi, Domenico Viggiano, Luca Voltolini

**Affiliations:** Thoracic Surgery Unit, University Hospital Careggi, Largo Brambilla, 1, 50134 Florence, Italy

**Keywords:** VATS lobectomy, Learning curve, Education, Minimal invasive surgery, Thoracic surgery

## Abstract

**Background:**

The development of a video assisted thoracic surgery lobectomy (VATS-L) program provides a dedicated surgical team with a recognized learning curve (LC) of 50 procedures. We analyse the results of our program, comparing the LC with subsequent cases.

**Methods:**

From June 2012 to March 2015, we performed *n* = 146 VATS major pulmonary resections: *n* = 50 (Group A: LC); *n* = 96 (Group B). Pre-operative mediastinal staging followed the National Comprehensive Cancer Network guidelines. All procedures were performed using a standard anterior approach to the hilum; lymphadenectomy followed the NCCN recommendations. During the LC, VATS-L indication was reserved to clinical stages I, therefore evaluated case by case.

**Results:**

Mean operative time was 191 min (120-290) in Group A and 162 min (85-360) in Group B (*p* <0,01). Pathological T status was similar between two Groups. Lymphadenectomy included a mean of 5.8 stations in Group A and 6.6 in Group B resulting in: pN0 disease: Group A *n* = 44 (88 %), Group B *n* = 80 (83.4 %); pN1: Group A *n* = 3 (6 %), Group B *n* = 8 (8.3 %); pN2: Group A *n* = 3 (6 %), Group B *n* = 8 (8.3 %). Conversion rate was: 8 % in group A (*n* = 4 vascular injuries); 1.1 % in Group B (*n* = 1 hilar lymph node disease). We registered *n* = 6 (12 %) complications in Group A, *n* = 10 (10.6 %) in Group B. One case (1.1 %) of late post-operative mortality (90 days) was registered in Group B for liver failure. Mean hospital stay was 6.5 days in Group A and 5.9 days in Group B.

**Conclusions:**

We confirm the effectiveness of a VATS-L program with a learning curve of 50 cases performed by a dedicated surgical team. Besides the LC, conversion rate falls down, lymphadenectomy become more efficient, indications can be extended to upper stages.

## Background

Since its introduction in 1991 [1], video assisted thoracic surgery lobectomy (VATS-L) for non-small cell lung cancer (NSCLC) has evolved to become a safe and effective alternative to the conventional thoracotomy approach [2, 3]. VATS-L, compared with lobectomy by thoracotomy, is associated with a shorter length of stay, less postoperative pain, preserved pulmonary function, fewer postoperative complications and better compliance with adjuvant chemotherapy [4–6]. Despite these advantages, recent data from European Society of Thoracic Surgeon database demonstrated that thoracotomic lobectomy is still the procedure of choice for surgical treatment of NSCLC and only 10 % (2721 vs 26051) of all procedures from 2007 to 2013 were performed with a minimally invasive approach [7]. Even if this circumstance may be related more to a cultural background, many authors report VATS-L as a more complex and time consuming procedure, with potentially serious intra-operative complications, moreover questioning its oncologic value particularly during the learning curve (LC) period. In this setting, many surgical teams could be demotivated in pursuing a VATS-L program. The length of a VATS-L LC has been established in 50 procedures [8]. However, several factors can affect duration and efficacy of the LC period: the experience in other complex VATS procedures and/or in standard open major pulmonary resections; the selection of a dedicated surgical team; the opportunity to concentrate the LC procedures within a short period and, last but not least, the development of a proctored and stepwise program [9–11].

In our series we analysed surgical and oncological outcomes (mortality, morbidity, hospitalization, operative time, type of resections, safety and effectiveness of mediastinal lymph node dissection and intra-operative staging) of a VATS-L program comparing the first 50 patients, representing our LC, with subsequent cases.

## Methods

Our institutional review board granted approval and waived the requirement for specific informed consent for this retrospective study. This is a retrospective study using a prospective database of consecutive patients who underwent VATS major pulmonary resections (VMPR) for NSCLC at our institution (Thoracic Surgery Unit, University Hospital Careggi, Florence) from June 2012 to March 2015.

Each patient was pre-operatively evaluated by computed tomography (CT) scan, positron emission tomography (PET) scan or PET/CT, pulmonary function test and bronchoscopy. Cervical video-mediastinoscopy (VM) was performed according to National Comprehensive Cancer Network (NCCN) guidelines (v. 2.2010): T > 3 cm, cN1 disease, central location [12]. VM was planned as a same-day surgical procedure, with frozen section (FS) analysis of lymph node biopsies in order to decide whether to proceed to lobectomy or to abort. A dedicated team, composed by two staff surgeons and two residents, was identified as in charge for the VATS-L program; team members were selected on the basis of a previous experience with other complex VATS procedures and a previous attendance at qualified training course on VATS-L. For the very first procedures (i.e. 10 cases), the two staff surgeons worked together, as first surgeon and first assistant. After this period, the team was composed by one staff surgeon and by the residents. Only in selected cases, the second staff surgeon was involved in the procedure. After completion of the LC, other staff surgeons of the division were invited to gradually join the program. The anaesthesiological team was composed by two staff anesthesiologists, with experience in the field of thoracoscopic procedures.

The first 50 cases of VATS-L (LC –Group A) were selected on the criterion of NSCLC at clinical Stage I, without endobronchial involvement at pre-operative bronchoscopy. Since the VATS-L program start-up, every case with these features referred to our division, was evaluated and eventually treated by the VATS-L team. After completion of the LC, also more advanced stages, considered as potentially resectable by VATS, were discussed and eventually included in the VATS-L program. Centrally located tumours, with the potential need of a bronchoplasty procedure, were always excluded.

All procedures were performed by a standardized three-port anterior approach, as previously described by Hansen et al [13]. No tissue retractor or rib spreading was used; in selected cases (e.g. obese patients) a wound protector (Alexis, Applied Medical, USA) was applied at the site of the utility incision. Lymph node dissection followed the NCCN (version 2.2010) recommendations [12]: “minimum of 3 N2 stations sampled or complete lymph node dissection”. In case of conversion, the anterior utility incision was extended to an anterolateral thoracotomy. We used extensively paravertebral block with single injection of local anesthetics (ropivacaine 75-100 mg plus lidocaine 200 mg) in several different intercostal spaces associated with intravenous administration of paracetamol/acetaminophen or non-steroidal anti-inflammatory drugs (NSAIDs) in the post-operative period.

Our policy for removing chest tubes is to take out them in absence of air leak and less than 200 mL of liquid output in 24 h.

Group A and Group B were compared in terms of surgical results (type of resection, operative time, blood loss, chest tube duration, hospital stay, intra-operative complications and conversions, postoperative morbidity and mortality) and oncological results (histology, pTNM, lymphadenectomy).

Statistical analysis was performed using SPSS 16.0 software (SPSS Inc., Chicago, IL). Continuous variables are expressed as mean values ± SD or median and range. Categorical variables were analysed using χ-square test. Continuous variables were compared by Student’s *t* test. A *P* value <0.05 was considered statistically significant.

## Results

We prospectively recorded data from 146 scheduled VMPRs chronologically divided into two groups: the first 50 cases, representing the LC (Group A), and the subsequent 96 cases, considered as a control group (Group B). The whole program (Group A and Group B) was initiated and completed by the same surgical team.

Demographical and pre-operative data are depicted in Table 1. There were no differences in the demographic and clinic-pathologic factors between the two groups. Since the different inclusion criteria, induction treatments are represented only in Group B. Type of resections and oncological results are showed in Table 2. According to 2.2010 NCCN pre-operative mediastinal staging guidelines [12], *n* = 27/146 (18.5 %) patients underwent VM with FS analysis of bioptic samples and, if negative, VMPR was planned as a same-day surgical procedure. FS sections showed a 0 % of both, false positive and false negative results. About type of resection, we observed a predominance of upper lobe lobectomies, equally distributed into the two groups. In Group B we performed a higher number of major lung resections different from lobectomies: 6.3 % (*n* = 6) versus 2 % (*n* = 1) in Group A. Particularly, in Group B we had: *n* = 1 (1.1 %) superior bilobectomy, for a double lesion of the upper and middle lobe, clinically N0; *n* = 1 (1.1 %) left pneumonecotmy, again for a double lesion involving respectively the upper and lower lobe, with endobronchial lesion at the level of the interlobar carina. Both groups were balanced for NSCLC histology. About pTNM, due to a selection bias, we obviously observed a prevalence of early stages (78 % stage I, 13 % stage II) in Group A, even if without statistically significance. Due to widening of surgical indications, in Group B we operated more advanced stages (T4 lesions in *n* = 3 patients).

**Table 1:** Demographical and pre-operative data

Variables	Group A (LC)	Group B	*P*
Age (years)	67 ± 7,1	66 ± 9,5	NS
Sex male	25 (50 %)	57 (59 %)	NS
Co-morbid disease			
Hypertension	23 (46 %)	53 (55,2 %)	NS
Heart disease	9 (18 %)	20 (20,8 %)	
Diabetes	1 (2 %)	4 (4,2 %)	
COPD	5 (10 %)	11 (11,4 %)	
Current smokers	18 (36 %)	34 (35,4 %)	
Neo-adiuvant treatment	0 (0 %)	6 (6,3 %)	
Pulmonary function			
FEV1%	76 %	70 %	NS
DLCO%	75 %	69 %	

*NS* not significant

**Table 2:** Pre-operative video-mediastinoscopy, type of VATS major pulmonary resection, final histological diagnosis, p-stage and intra-operative lymphadenectomy

Variables	Group A (LC)	Group B	*p*
Procedure			
Video-mediastinoscopy with frozen sections	11 (22 %)	16 (16,6 %)	NS
RUL	15 (30 %)	33 (34,3 %)	
ML	3 (6 %)	4 (4,2 %)	
RLL	10 (20 %)	18 (18,7 %)	
LUL	16 (32 %)	15 (15,6 %)	
LLL	5 (10 %)	20 (20,8 %)	
Upper bilobectomy	0	1 (1,1 %)	
Left Pneumonectomy	0	1 (1,1 %)	
Segmentectomy	1 (2 %)	4 (4,2 %)	
Histology			
Adenocarcinoma	33 (66 %)	71 (74 %)	NS
Squamous cell carcinoma	12 (24 %)	15 (15,6 %)	
Others	5 (10 %)	10 (10,4 %)	
• Large cell carcinoma	1	3	
• Carcinoid	4	7	
Pathological T stage			
1a	26 (52 %)	41 (42,7 %)	NS
1b	17 (34 %)	21 (21,8 %)	
2a	6 (12 %)	18 (18,8 %)	
2b	0	6 (6,3 %)	
3	0	7 (7,3 %)	
4	1 (2 %)	3 (3,1 %)	
Pathological N stage			
0	44 (88 %)	80 (83,4 %)	NS
1	3 (6 %)	8 (8,3 %)	
2	3 (6 %)	8 (8,3 %)	
Number of nodal station removed	5,78 ± 0,9	6,55 ± 1,1	<0,01

*NS* not significant

About results of VATS intra-operative lymphadenectomy, the mean number of hilar and mediastinal dissected lymph node stations statistically differ between the two groups (Table 2).

**Fig. 1:**
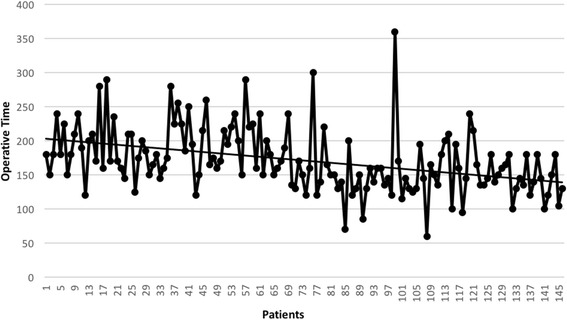
Operative time for each patient

**Fig. 2:**
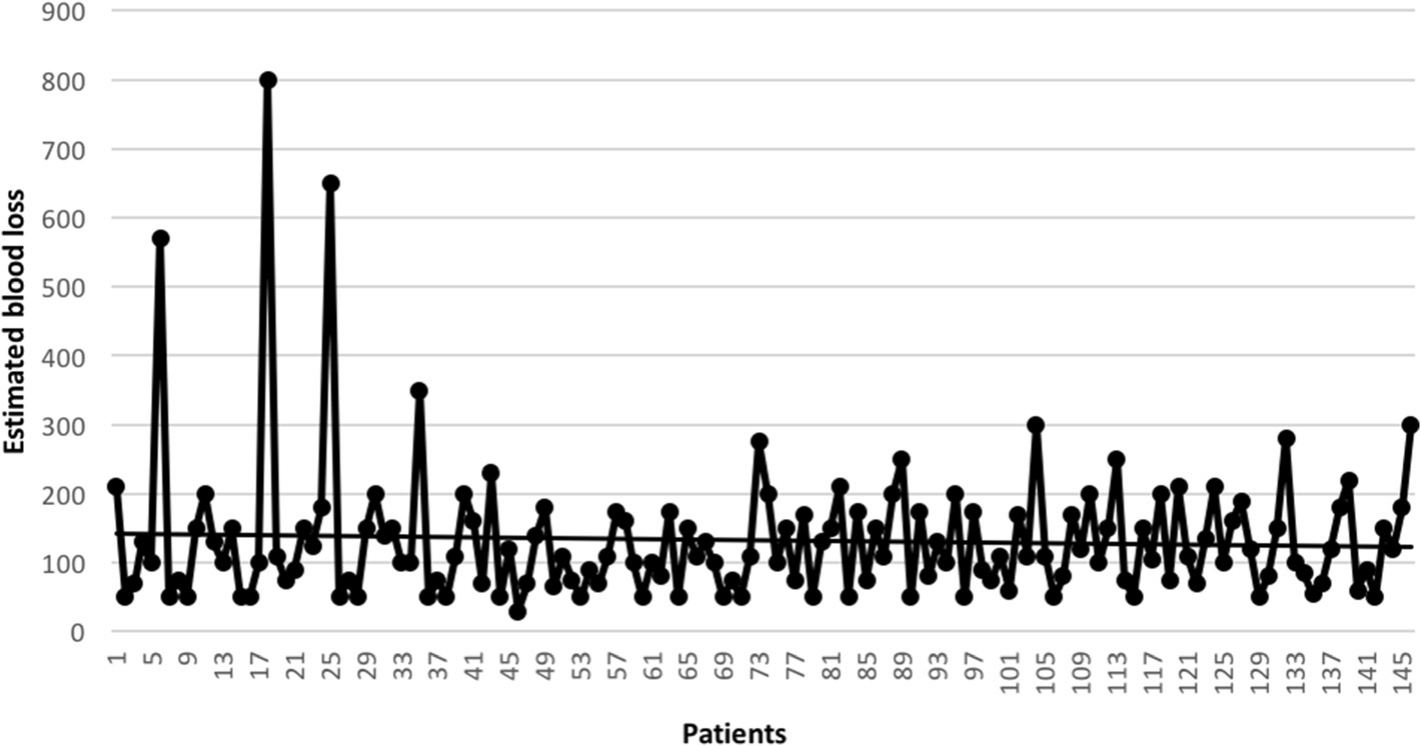
Intraoperative estimated blood loss, expressed in ml, for each patient

Surgical data and post-operative outcomes are showed in Table 3. The operative time (detailed for each patient in Fig. 1) was statistically significantly shorter in Group B. There was a not statistically significant difference between the estimated blood loss in the two groups (Fig. 2). The conversion rate was statistically significantly lower in Group B than in Group A (1 % *vs* 8 % respectively, *p* = 0,02). Conversions were due to vascular injuries in all cases of Group A: they were *n* = 3 (6 %) bleeding (arterial bleeding *n* = 2, 4 %; venous bleeding *n* = 1, 2 %), of which *n* = 2 (4 %) considered as “bleeding not safely manageable by VATS” and *n* = 1 (2 %) as a “life threatening bleeding”. Finally, *n* = 1 (2 %) conversion in Group A was due to an incorrect transection of the main left pulmonary artery instead of the upper mediastinal branch during a left upper lobe lobectomy; after conversion, this patient was managed by an end to end anastomosis of the vascular stumps. In Group B, *n* = 1 (1.1 %) conversion was due to an hilar lymphadenopathy. Complication rates were similar between the two groups. We registered *n* = 2 major complications, both in Group B: *n* = 1 acute lung injury (ALI); *n* = 1 acute liver failure, evolved with a multiple organ failure (MOF). This last patient represented the *n* = 1 case of mortality in Group B. Chest drain duration and hospital stay (Group A 6,5 ± 2,5 vs Group B 5,9 ± 1,9; range 4-12 vs 4-28) were similar between the two groups; *n* = 68 (46 %) patients were discharged within the fifth p.o. day. Our policy in removing chest tubes is to take out them when the drained is less of 200 ml in the last 24 h and this can affect directly the hospitalization.

**Table 3:** Operative, post-operative data, morbidity, mortality

Variable	Group A (LC)	Group B	*p*
Operative time	190,9 ± 41,4	162 ± 47,4	< 0,01
Estimated blood loss	154 ± 152	122 ± 69	NS 0,08
Conversion rate	4 (8 %)	1 (1.1 %)	0.04
Chest tube duration	5,4 ± 1,8	5,1 ± 1	NS
Hospitalization	6,47 ± 2,5	5,92 ± 1,9	NS
Post-operative complications	6 (12 %)	10 (10.6 %)	NS
• Bleeding requiring transfusions	1 (2 %)	1 (1.1 %)	
• Prolonged air leak	1 (2 %)	4 (4.2 %)	
• Atrial arrhythmia	1 (2 %)	3 (3.1 %)	
• Acute lung injury	0	1 (1.1 %)	
• Liver failure and Adult Respiratory Distress Syndrome	0	1 (1.1 %)	
• Pneumonia	1 (2 %)	0	
• Recurrent laryngeal nerve palsy	1 (2 %)	0	
• Hoarseness with normal vocal cord motility	1 (2 %)	0	
Mortality (60 days)	0 (0 %)	1 (1.1 %)	NS

*NS* not significant

## Discussion

VATS-Lobectomy, is recognized to be associated with many advantages compared with lobectomy by thoracotomy [2–4, 14, 15]. Recent analysis of postoperative outcomes performed on both, single institutional series and official database, proposed VATS-L as to be superior in terms of length of stay, postoperative pain, preserving pulmonary function, postoperative complications and compliance with adjuvant chemotherapy when compared to open lobectomy. Despite a 20 years period of development and published reports of thousands of cases, VATS-L remains a technique adopted by a minority of thoracic surgeons, at least when we look to national database; moreover, VATS-L have experienced only in the last years a significant increase of its uptake [7]. VMPRs are still considered complex and demanding procedures characterized by the need of a fine dissection of delicate and vulnerable vascular structures at risk for potential severe and life threatening bleedings. The LC period is considered the period more at risk for these complications. The adequacy of the oncological result is the other side of the coin. Even if several authoritative authors [14, 16, 17] demonstrated the efficacy of VATS-L in terms of oncological results and validity of intra-operative staging, the issue is still debated. Particularly, the safety and effectiveness of VATS mediastinal lymphadenectomy outside specialty centers or during the LC period is considered a critical point; in these settings, some technical difficulties, such as lymph node exposure and en bloc dissection, could be time consuming or considered as risky, thus leading to an oncologically inadequate result. Actually, an incomplete mediastinal lymph node dissection/sampling in NSCLC, may result in an incorrect staging and patients would be denied significant chances of cure (i.e. adjuvant chemotherapy in stage IIA and higher). For these reasons VMPRs are not evenly spread among thoracic surgeons, despite obvious advantages.

We planned our VATS-L program in 2011, based on our previous experience with minimally invasive technique. We decided to select two staff surgeons and two residents, on the basis of their skills in complex VATS procedures different from VMPRs. This was done not only to take advantage from the previously acquired skills, but mainly to avoid cultural prejudices against VATS-L, potentially present in a heterogeneous (with regard to age and cultural background) surgical division. Therefore, they were involved in qualified VATS-L training courses. We started our VATS major pulmonary resection program in June 2012, achieving the fifty procedures of the expected LC in about 12 months, with a number of at least 4 lobectomies per months; this number allowed the attainment and maintenance of learned skills. There are many other human factors that may influence the LC and consequently the success of a VATS-L program. First, the thoracoscopic experience of the whole surgical team, including anesthesiologists and nurses. The surgeons should have performed a considerable number of VATS procedure (e.g. wedge resections, mediastinal procedures, debridement of pleural empyema) but also should have a solid thoracotomic background, helpful to prevent and to solve any intra-operative complication. Another key point is the attendance of qualified courses in VATS-L or in centers with a high VATS-L volume. Creating a motivated and supportive team is crucial.

From the technical point of view, our series differs from other authors [9–11], even in the LC, for the prevalence of upper lobectomies (54 %: right upper lobectomy *n* = 48/32 % and left upper lobectomy *n* = 31/21 %) that are considered technically harder than lower lobectomies, thus influencing our operative time and conversion rate at least at the beginning. During the LC we strictly adopted the inclusion criterion of clinical Stage I NSCLC, in order to minimize the risk of complications. However, an incompletion fissure at pre-operative scan was not considered a controindication, as well as we did not start including only lower lobe lobectomies; on the contrary, we experienced lower lobectomies with incomplete/absent fissure as the more complex procedures. As it happens for all surgical procedures, their repetition and re-iterativity allows a shorter and effective LC. By shifting to our VATS-L program all the suitable Stage I NSCLCs, we wanted to increase the frequency of VATS-L during the LC period, so helping the surgical team in its growth. Clearly the surgical volume of the center, first affect the length of the LC; our 500 (approximately) procedures per year ensured us a sufficient volume. A surgical LC can be considered as completed when parameters and results are stable, reaching a steady state and becoming comparable with literature data. Operative time, conversion and complication rate, hospital stay, oncologic adequacy and number of dissected lymph node stations are considered critical data during a VATS-L program. Our study showed that: 1) our LC results are in line with literature; 2) performance indicators can improve with increasing experience, maintaining oncological adequacy while enlarging indications (Table 3). Actually, operative time and conversion rate were significantly lower after the advised fifty procedures, whereas we noted a statistically significant improvement in the number of dissected lymph node stations. About lymph node dissection, the fear of not being effective, overall during the LC period, has proved to be baseless. Even if lymphadenectomy improves after completion of the LC, as we expected, however a mean number of dissected lymph node stations >5 demonstrates VATS effectiveness also during the LC.

Our policy for chest tube removal (no air leak; <200 ml/24 h) is quite common. The incidence of prolonged air leak is low, about 3 %, and so does not influence the mean hospital stay. However our chest drain duration and hospitalization is longer than another VATS-L series both, during learning curve and after its completion [16]. These data reflect more the presence of different cultural backgrounds in our surgical team than a careful chest tube management policy. Our team is heterogeneous and consists of surgeons who have embraced the “minimally invasive philosophy” and other “traditional” surgeons for which is not possible and unthinkable an early chest tube removal and patients discharge before the fourth post-operative day.

Data from literature showed conversion rates to thoracotomic lobectomy in a range between 2 and 10-12 % [2, 14, 17]. Causes of conversion are various and reported as technical problems (e.g. poor visualization, instrumentation malfunction), anatomical problems (calcified peri-arterial lymph nodes, absent fissure, adhesions), intra-operative complications (massive bleeding from vascular injury) and oncological situation (invasion of chest wall, invasion of vascular structures, intra-operative unexpected N2 status, centrally located tumor, sleeve resection). Some of these factors are absolutely random and stochastic and surgeon can only prevent these problems with an accurate pre-operative study including patients’ characteristics, radiological and endoscopic findings that could anticipate intra-operative critical technical aspects. A relative contraindication to VMPRs is considered the presence of hilar and perivascular calcification, since it may lead to a technical demanding vascular dissection with an increased risk of major bleedings; we faced this condition in 1.1 % of cases in Group B. In a large recent series Villamizar et al. [18], reported an overall conversion rate of 4 % (36/916), caused in 21 patients (2 %) by an intra-operative bleeding; they also found a significant relation with the presence of positive lymph node stations (*n* = 11 conversions in 153 clinical N+ patients/7.2 % vs *n* = 25 in 763 clinical N0 patients/3.3 %). In our series, in Group A we observed three conversions (6 %) due to “bleeding not safely manageable by VATS”; in our opinion this datum reflects a low experience in bleeding management by VATS and is influenced also by an attitude to an immediate conversion rather than an attempt of repair (quite normal during the LC period). However, our series demonstrated that an emergency conversion is not a frequent event (1/146, 0.7 %), even in the early LC (1/50, 2 %). Actually, we consider the wrong transection of the main pulmonary artery, performed during a left upper lobe lobectomy, as the worst complication of the whole series. Obviously, with the increased proficiency, we believe it is ethically correct to propose VMPRs even in patients with co-morbid disease (e.g. low pulmonary reserve), previously treated with neo-adjuvant chemotherapy or with surgically treatable advanced disease (T3 or T4). In our experience we started to “extend” indications after the conclusion of the LC, when the acquired skills and results made us more confident in our technical skills: in Group B we performed 6.3 % of VMPRs after induction chemotherapy and we pushed the indications beyond the limit of a standard lobectomy (pneumonectomy 1.1 %, bilobectomy 1.1 %, chest wall resection 1.1 %), without increasing procedure-related complications or decreasing oncological adequacy.

The critical point, widely debated in literature, is the oncologic adequacy of VMPRs. Watanabe et al. in 2005 [19] and more recently Stephens et al. [20] compared lymph nodes number and upstaging between VATS and open lobectomy for NSCLC and they found no significant differences [9, 16, 19–21]. Other studies demonstrated that the LC had no negative impact on lymph node number or dissected nodal stations, remaining always oncologically effective [9]; our results are in line with this literature with no differences before and after accomplishment of the LC (Table 2).

To better understand results, we believe that collect, analyze and compare data is basic; moreover, the opportunity to compare your results with other reliable data from official database has an added value. In January 2014, Crisci R. (University of L’Aquila, Italy), bringing together the Italian centers performing VMPRs, created a VATS-L Italian community (www.vatsgroup.org), in order to promote the diffusion of VATS-L, collect and analyze surgical and oncological data coming from the participating centers. We think that this step will lead to a professional growth and expansion of the Italian VATS-L community.

Our study had some limitations: 1) is a single institution series, retrospectively analyzed in a short period and with limited oncological follow-up; 2) obviously, during the LC we selected patients with early stage lung cancer and this issue can be interpreted as methodological bias; 3) furthermore there are not standardized parameters to evaluate and quantify the surgeons’ performance, proficiency and efficiency.

## Conclusions

We conclude that the safety and effectiveness of a VATS-L program with a learning curve of 50 cases performed by a dedicated surgical team has been confirmed by our study. Besides the LC, conversion rate falls down, lymphadenectomy become more efficient, indications can be extended to upper stages.
